# Engaging people with lived experience on advisory councils of a national not-for-profit: an integrated knowledge translation case study of Heart & Stroke Mission Critical Area Councils

**DOI:** 10.1186/s12961-022-00863-w

**Published:** 2022-05-23

**Authors:** Krystina B. Lewis, Nedra Peter, Heart & Stroke Foundation of Canada, Christine Faubert, Mary Elizabeth Harriman, Patrice Lindsay, Anne Simard, Cindy Yip, Ian D. Graham, Anita Kothari

**Affiliations:** 1 School of Nursing, Faculty of Health Sciences, University of Ottawa, University of Ottawa Heart Institute, Ottawa, Canada; 2 Schulich School of Medicine and Dentistry, Western University, London, Canada; 3 School of Epidemiology and Public Health, School of Nursing, Ottawa University, Ottawa Hospital Research Institute, Ottawa, Canada; 4 School of Health Studies, Western University, London, Canada

**Keywords:** Integrated knowledge translation, Knowledge users, Knowledge mobilization, Strategic planning, Advisory committee, Health systems, Health research funder, Third-sector organization, Patient engagement, Knowledge co-creation

## Abstract

**Background:**

In 2018, the Heart and Stroke Foundation of Canada transformed its approach to organizational strategic planning and priority-setting. The goal was to generate impact from bench to bedside to community, to improve the health of Canadians. It engaged researchers, clinician scientists, health systems leaders, and community members including people with lived experience (PWLE) on six Mission Critical Area (MCA) councils, each of which was co-chaired by a researcher or clinician scientist and a person with lived experience. Together, council members were tasked with providing advice to Heart & Stroke about the most relevant and impactful priorities of our time. The aim of this research was to explore the value of the MCA councils to Heart & Stroke, and to council members themselves. The research questions focused on understanding the process of managing and participating on the councils, the challenges and outcomes.

**Methods:**

Using an integrated knowledge translation approach, we conducted a case study with developmental evaluation over a 2-year time period (2018–2020). We collected qualitative data from various sources (Heart & Stroke team responsible for managing the councils, council co-chairs, council members, and key informants). We collected documents and analysed them for contextual background.

**Results:**

Participants noted that the MCA councils continuously evolved over the 2 years in various ways: from an uncertain direction to a concrete one, better integrating the voice of PWLE, and increased cohesiveness within and across MCA councils. This evolution was achieved in parallel with successes and challenges at three levels: the MCA councils and its members, Heart & Stroke, and Canadians. The MCA councils were disbanded in 2020, yet learnings, developments, initiatives and established partnerships remain as their legacy.

**Conclusions:**

Heart & Stroke’s intended objectives for the MCA councils, to promote engagement and dialogue among community members including PWLE, clinician scientists, and researchers, and to provide advice into Heart & Stroke’s strategic renewal process, were achieved. This collaborative structure and process for PWLE engagement within a community of multidisciplinary clinician scientists and researchers is possible yet requires flexibility, commitment to stakeholder relationship management, and considerable resources. These findings may be helpful for other not-for-profit and funding organizations interested in engaging the public and other stakeholders into their organizational activities.

**Supplementary Information:**

The online version contains supplementary material available at 10.1186/s12961-022-00863-w.

## Background

The Heart and Stroke Foundation of Canada (H&S) is a Canadian charity dedicated to policy and advocacy, public awareness and education, and the funding of research on heart disease and stroke. In 2017–2018, H&S revitalized its approach to organizational planning and priority-setting for the breadth of its mission activities (e.g. education, awareness, policy, health systems change, best practice and research), with the goal of generating prompt impact from the research it funds to improve the health of Canadians. The H&S created six Mission Critical Area (MCA) councils, based on a taxonomy of diseases/conditions in the areas of heart and brain health that represent the greatest burden on Canadians’ health, the economy and society (Table 1). The MCA councils gathered clinician scientists, researchers, health system leaders and people with lived experience (PWLE) as members. Each council was co-chaired by a researcher or clinician scientist and a person with lived experience. Specifically, the MCA councils were tasked with providing advice to H&S on the prioritization of the most important emerging issues, trends and focus areas with the most potential for impact as related to the three pillars of H&S’s mission: *Promote health. Save lives. Enhance recovery.*

**Table 1:** MCA councils’ objectives and areas

Objectives of the MCA councils
Promote engagement of, and dialogue among, individuals with lived experience and those from multidisciplinary scientific and clinical communities
Provide advisory input on the current state, emerging issues and trends, and near- and long-term areas of focus for H&S within the six MCAs
Foster integration and strengthen the collective knowledge base in research, patient engagement, knowledge exchange, education, system change, advocacy, health promotion and government relations, with the overall goal of improving health outcomes

MCAs	Description
Stroke	Interruption in blood flow to the brain
Heart failure	Inability of the heart to pump blood effectively due to damage or weakness
Vascular cognitive impairment	A range of memory or thinking deficits, from mild problems (i.e. forgetfulness, problem-solving difficulties) to vascular dementia, caused by a lack of oxygen to blood vessels in the brain
Heart rhythm	Irregular heart rhythms due to disruption of the normal electrical impulses of the heart (i.e. sudden cardiac arrest, atrial fibrillation and other arrhythmias)
Coronary artery and vascular disease	Narrowing or blockage of the coronary arteries (i.e. heart attack, angina) and abnormalities of arteries or veins that reduce blood flow between the heart and the body, leading to heart disease
Structural heart disease	Defects or abnormalities compromising the function of heart chambers and valves

### Co-creation of knowledge to narrow the knowledge-to-action gap

H&S’s ambition to reduce the knowledge-to-action gap is shared by many. Governments, research funders, universities and other stakeholders are also concerned about waste in healthcare research given the billions of dollars spent, with findings often not being used in health policy or practice [1]. When they are, a significant time lag exists between the availability of findings and their subsequent uptake in practice or policy, highlighting missed opportunities to improve people’s health [2]. One proposed solution is knowledge co-creation. According to Greenhalgh and colleagues (2016), “co-creation—collaborative knowledge generation by academics working alongside other stakeholder—reflects a ‘Mode 2’ relationship (knowledge production rather than knowledge translation) between universities and society…. [and] is widely believed to increase research impact” [3]. The authors list three key principles to its success, which include “(1) a systems perspective (assuming emergence, local adaptation, and nonlinearity); (2) the framing of research as a creative enterprise with human experience at its core; and (3) an emphasis on process (the framing of the program, the nature of relationships, and governance and facilitation arrangements, especially the style of leadership and how conflict is managed)” [3]. When researchers and stakeholders come together to co-create relevant and usable knowledge, impact on practice, policy, health systems and societal outcomes is greater and more timely, narrowing the knowledge-to-action gap [4, 5].

Historically a function of senior leadership, organizational strategic planning and priority-setting processes are increasingly including a wide range of stakeholders as equal partners on teams [6]. The goal of engaging a diverse group of stakeholders is to create change that is meaningful and aligns with the broader community’s values, whether by setting new priorities or improving health services, for example [7]. This type of engagement has become increasingly preferred over passive feedback from community members where they do not have a say in setting the agenda, how their contributions are interpreted, and whether even integrated into health services. The use of engagement models promotes the avoidance of tokenistic inclusion, and ensures that the community voice is actively, fairly and continuously represented in the most meaningful phases of the process [8]. These inclusive practices lead to priorities, programmes and research that are more inclusive, robust and strategic [9]. H&S deliberately involved multiple stakeholder groups, including PWLE, in their organizational strategic planning and in setting mission priorities. Given that PWLE’s engagement in organizational strategic planning and priority-setting is in its infancy, there is a need for evidence to enhance our understanding about what works (and what does not) in achieving and sustaining productive PWLE engagement with others from multidisciplinary scientific and clinical communities [10].

### Integrated knowledge translation team: positionality

H&S was interested in evaluating the benefits and challenges of driving organizational strategic priorities in this novel way. By prospectively studying the process, H&S hoped to optimize input from MCA councils and identify new ways to approach the knowledge-to-action gap through its research funding. H&S reached out to members of our team, university-based researchers (AK, IDG), to suggest the MCA councils initiative as an interesting case to study given our interest in studying the process of integrated knowledge translation (IKT) and co-creation. Our research team consisted of four university-based researchers interested in understanding how community members, including PWLE, and caregivers, clinician scientists, researchers and health system leaders, jointly co-create and mobilize various types of knowledge (e.g. current evidence and data trends, knowledge from the lived experience of heart and stroke conditions, contextual knowledge of the organization and sociopolitical influences, and prioritization of research priorities). We were also outsiders to the MCA council process and could therefore provide an independent and impartial examination of the process, experience and impact of the councils. Together with H&S, we committed to applying an IKT approach to the study, an approach that has been widely promoted and used in health research in Canada [11]. Kothari et al. [12] define IKT as “a model of collaborative research, where researchers work with knowledge users who identify a problem and have the authority to implement the research recommendations”. Hence, every step of this research was shaped through iterative discussions with H&S: from the generation of the research questions, to data collection planning and execution, to the interpretation of the findings, and the writing of the final report.

### Aim

The overarching aim of this research was twofold. First, to understand how advisory councils composed of PWLE and others work collaboratively to inform organizational mission priorities. And second, to understand the value of the MCA councils to H&S and to council members themselves. Specifically, the research questions focus on understanding the process of managing and participating on the councils, and the related challenges and outcomes emerging from this approach to engagement:

1. What was the experience of leading, managing and participating on the MCA councils?
2. What were the challenges resulting from this approach to engagement?
3. What were the outcomes arising from this approach to engagement?

## Methods

We conducted a qualitative case study guided by Stake’s methodology [13]. We selected this approach given its inductive and flexible nature, its attention to context and its alignment with the constructivist view we held as we embarked on this research with the H&S team. Given the prospective nature of this research, we further informed this work using a developmental evaluation approach [14]. This approach is well suited to innovative programmes such as the MCA councils because it permits the provision of real-time feedback (to H&S) and allowed us to be responsive to the dynamic environment in which the MCA councils were being managed [14]. In other words, we were able to adapt our research approach throughout the process (e.g. data sources, data collection methods) to best answer our research questions. This flexibility was integral to honouring our IKT approach with H&S knowledge users and to the evolving nature of the MCA councils. We used the consensus standards for the reporting of organizational case studies [15] and the Standards for Reporting Qualitative Research [16] to guide the reporting of this study.

### Setting and participants

Participants included researchers, clinician scientists, health systems leaders and community members, including PWLE. Participants were situated at multiple levels within the MCA councils and H&S, including council members, council co-chairs, H&S staff and key informants at senior levels and external to the organization. MCA council activities took place over 2 years by teleconference and in-person meetings with study data collection activities interspersed throughout. We provide a detailed account of study activities in Table 2.

**Table 2:** Data collection and timeline

Year 1			
Date	Data source	No.	Method
2017–2018	Documents	20	Document review
July–November 2018	MCA council co-chairs	7^a^ Clinician scientist or researcher *n* = 3 Community member, including PWLE, *n* = 4	Interviews
January–February 2019	MCA council members	25^b^ Clinician scientist or researcher *n* = 15 Community member, including PWLE, *n* = 10	Focus groups
March 2019	H&S team	4	Focus group

Year 2			
Date	Data source	*n*	Method
2019–2020	Documents	7	Document review
14 January 2020	MCA council co-chairs	11 Clinician scientist or researcher *n* = 7 Community member, including PWLE, *n* = 4	Focus group
14 January 2020	MCA council members	N/A	Distribution of PEIRS questionnaire
12 February 2020	H&S team	4	Focus group
14 February 2020	Key informant	1	Interview
18 February 2020	Key informant	1	Interview
15 March 2020	Key informant	1	Interview
16 March 2020	Key informant	1	Interview

*H&S* Heart & Stroke, *MCA* Mission Critical Area, *N/A* not applicable, *PEIRS* Patient Engagement in Research Scale, *PWLE* people with lived experience

^a^Representing five of six MCA councils

^b^Representing six of six MCA councils

### Data collection

To obtain a holistic understanding of the MCA councils, we collected data using various methods including focus groups, individual interviews, and documents over a 2-year time period (2018–2020). Data sources included (1) the H&S team responsible for the organization of the MCA councils, (2) co-chairs of the MCA councils, (3) members of the MCA councils, (4) key informants from the H&S and affiliates who were identified by the H&S team as having important and valuable perspectives on the impact of the MCA councils, and (5) relevant documents about the H&S and MCA councils (Table 3). KBL, NP and AK shared the task of conducting the interviews and focus groups. Many were conducted by telephone, with others conducted face to face during H&S’s annual meeting of its most senior advisory body, the Council on Mission: Priorities, Advice, Science and Strategy (CoMPASS). A survey based on the participant version of the Public and Patient Engagement Evaluation Tool [17] was distributed in early 2020, but due to the COVID-19 pandemic, the survey was not administered and was ultimately cancelled.

**Table 3:** Data sources

Data source (eligible participants)	Description of members	Justification for inclusion	Topics of interest discussed during data collection
(1) H&S team	H&S staff who collaborated on this research and worked directly with the MCA councils	To gain an understanding of the organization’s experiences of leading and managing the councils	-MCA council management—MCA council support -Interactions with the councils and members -Lesson learned from this mechanism of engagement
(2) MCA councils	Researchers, clinician scientists, health systems leaders and community members, including PWLE	To gain an understanding of participating on the MCA councils	-MCA council functioning -Members’ level of engagement -Knowledge sharing -Knowledge construction -MCA council support provided by H&S -Challenges -Outcomes
(3) Council of MCA co-chairs	Each MCA council was led by two co-chairs, a clinician scientist or researcher and a community member (including PWLE), who were also a part of the council of co-chairs, which operated like a community of practice. Co-chairs learned from each other about challenges and innovations emerging from the individual councils they led	To gain the perspective of the individuals leading the MCA councils, and understand how knowledge sharing occurred within and across councils	-Evolution of the MCA councils -Challenges and opportunities -Opportunities for improvement -Outcomes -Lessons learned
(4) Key informants	Key informants internal to the H&S, and affiliates, who were identified by the H&S team as having important and valuable perspectives on the impact of the MCA councils	To gain an external perspective of the value and impacts of the MCA councils to the H&S and beyond	-Their experiences with the MCA councils or council-related activities -Anticipated and unanticipated benefits and consequences -General observations
(5) H&S (and MCA council-specific) documents	Documents related to the creation and maintenance of the MCA councils, which the H&S team identified as important and relevant to our study	To provide contextual and historical information within which to frame the case	Questions we asked ourselves when going through these documents: -Purpose of MCA councils -Supporting the MCA councils -Planning activities -Outcomes

*H&S* Heart & Stroke, *MCA* Mission Critical Area, *PWLE* people with lived experience

### Data analysis

The analysis plan was collaboratively discussed with the research team. Ethical requirements insisted that H&S knowledge users be at a distance from the analysis to maintain confidentiality of MCA co-chairs and council members. Hence, the data analysis was executed by three PhD-prepared university-based researchers (KBL, NP, AK) with experience in qualitative analysis. The emphasis of the analysis was on revealing a holistic account of the MCA councils from various perspectives. We illustrate this global perspective by sharing quotations drawn from individual MCA council members, co-chairs, H&S staff and key informants, without referencing their specific role. We analysed participant responses through thematic analysis to identify, analyse, organize, describe and report core themes based on our research questions. Using a process of inductive analysis, we noted meaningful segments of data and documented patterns and themes [18]. These were then clustered into emergent thematic areas. Documents were analysed for contextual background. As per an IKT approach, we discussed our perspectives and understandings of the data during team meetings and engaged in discussions to interpret findings with the H&S team. All recordings from interviews and focus groups were professionally transcribed, cleaned and de-identified. Transcripts and documents were stored in NVivo, a qualitative data management software.

### Ethical considerations

The Western University Research Ethics Board reviewed and approved this study (Project ID: 111043). All participants provided written informed consent. The research team worked diligently within the scope of the Canadian research tri-council ethics policy and related guidelines; for example, the team was careful about how quotes were presented to safeguard participants’ privacy.

## Results

In year 1, 25 MCA council members, seven co-chairs and four H&S team members participated in interviews and/or focus groups. In year 2, 11 co-chairs and the same four H&S team members participated in another focus group, with an additional four key informants interviewed. We collected a variety of internal documents in both year 1 (*n* = 20) and year 2 (*n* = 7), including agendas and minutes of meetings, presentations, policies, membership selection considerations, framework for dialogue, a description of members’ roles, and tangible examples of MCA council outputs (e.g. reports, grant submissions), and planning and organizational documents. As per case study research, we begin the results section by describing the context of the MCA councils. We then organize our findings according to our three research questions. We provide a summary of the results in Table 4.

**Table 4:** Summary of results

Research question	Thematic areas with supportive quotations
1. What was the experience of leading, managing and participating on the MCA councils?	Leading, managing and participating on the MCA councils was characterized by evolutions, which transpired in three distinct ways: (1) Shifting from an uncertain and unclear direction to concrete direction: *We’ve moved from uncertainty and this vague objective to actually coming out with some very concrete and strategic directions.* —MCA council co-chair (2) Better integrating the voice of PWLE: *I feel like now when we’re in conversation or we’re discussing this, everyone is just coming from a place of wisdom, not necessarily their role or their expertise or like it just feels like we’ve come to a much richer place because I think we’re listening to each other more fully and we are all sort of really listening for direction without worrying so much about the work we’ve done in the past or the experiences we’ve had in the past.* —MCA council member (3) Increasing cohesiveness within and across MCA councils: *We had created six bodies that would effectively reinforce that… It would cement the walls that we want to break down because people’s experience of health is holistic, yet the system is incredibly fragmented, and by having a process that was by definition fragmented in six groups, there were significant risks, so I said I’m quite happy to have six tables, but I want to make sure that the process will involve a chance for the six tables to come together, and I want to make sure that at the end of the day, the big priorities that come out are transcending.* —Key informant 1
2. What were the challenges resulting from this approach to engagement?	A combination of smaller, logistical challenges were amenable to change, while others were more difficult to resolve completely. They included: (1) Managing the councils and its membership: *…months going by and they weren’t as engaged. So to get them back engaged, that also takes time, and you’re also shortening a timeline period.* —MCA council co-chair (2) Lack of organizational structure in place to support the initiative: … *a very complex process in an organizational context that didn’t have the foundation to really support it.* —H&S team member (3) Terminating the MCA councils: *In year 2, we were very clear that this was the last time they were meeting as individual councils. And we really were like, you know, the mandate is ending, we gave lots of forewarning you know. Whether they really heard it or not or really believed it or not is always a different issue.* —H&S team member
3. What were the outcomes arising from this approach to engagement?	Taken together, the MCA council process generated greater than expected outcomes at three main levels: (1) The MCA councils: *We are very different people, we’ve been brought together but with very different perspectives and experiences. So I think that’s what makes it very rich, to bring all those people together who have very different, may have very different perspectives.* —MCA council member (2) The H&S: *…growing out of our Mission Critical Area councils but also kind of spreading our intent, our message, and kind of our voice in a global context.* —H&S team member (3) Canadians: *bring things together at a level that the focal point being disease areas that are broad, large and are well understood, in particular, by the public* —MCA council co-chair

*H&S* Heart & Stroke, *MCA* Mission Critical Area, *PWLE* people with lived experience

### The context: MCA council structure and process

The structure of the councils was based on the six heart and brain conditions that together represent the biggest burden on Canadians’ health, economy and society [19]. The selection of the 10–12 MCA council members per council was guided by selection criteria aiming to balance the geographical, cultural, demographics, skills, career stage and experience diversity of the councils. About half of each council were community members. Each council was co-chaired by a researcher or clinician scientist and a community member. The MCA councils were each tasked with examining the current science and gaps in their topic area, and providing advice to the H&S on emerging issues, trends and focus areas within the MCAs with the greatest potential for impact.

The initiation of the MCA councils in 2017–2018 occurred in a context of strategy renewal. This permitted the MCAs to inform and help shape a broader organizational planning and priority-setting process. Historically, H&S convened CoMPASS, the mission team’s most senior advisory body composed of approximately 35 Canadian researchers, clinicians and PWLE of heart disease and stroke, to inform its strategy. With a change in leadership at both the chief executive officer (CEO) and chief mission & research officer level during the first 6 months of the creation of the MCA councils, H&S evaluated and then renewed the vision of the MCA councils to find them a purpose anchored in a new strategy. H&S determined that the MCA councils would advise H&S on the “big priorities of our time”. From here, the MCA councils would, through structured discussion, identify condition- or disease-specific priorities as well as crosscutting priorities for the organization and the research it funds. The organization believed that the engagement of community member voices in this process could expand the richness and type of wisdom in discussions and saw the new structures as an innovative approach to priority-setting. The six MCA co-chairs were also integrated into CoMPASS.

A council of co-chairs was also created to encourage knowledge sharing across the six different MCA councils, allowing H&S to move to new, promising frontiers within and across the MCAs. Once formed, each council met through teleconference and face-to-face meetings to identify its priorities. The H&S team then synthesized these priorities across councils and identified commonalities and distinctions between them. These were further discussed and prioritized at a face-to-face meeting with all six MCA councils and CoMPASS leadership. The H&S team and the council of co-chairs synthesized the deliberations and refined the prioritized crosscutting themes, which were presented to and approved by the H&S Board of Directors in March of 2020 (see Table 5 for a list of the board-approved priorities). Each MCA council met for an initial engagement/working session on selected priority initiatives from March to June 2019. The MCA council mandate was closed at the face-to-face CoMPASS meeting in January 2020. Please refer to the Additional file 1 for a detailed timeline and account of the MCA council and H&S activities.

**Table 5:** Top five MCA priorities endorsed by the H&S Board in March 2020

Priority 1. The interconnections between heart and brain health
Priority 2. Women’s heart and brain health and Indigenous health
Priority 3. Precision health and individualized care
Priority 4. The patient’s journey to rehabilitation, recovery and optimal health
Priority 5. Upstream health promotion/prevention

### Experience of leading, managing and participating on the MCA councils

The MCA councils evolved in three distinct ways during its mandate. Specifically, participants noted that the MCA councils evolved by (1) shifting from an uncertain and unclear direction to a concrete one, (2) better integrating the voice of PWLE and (3) increasing cohesiveness within and across MCA councils.

#### Shifting from an uncertain and unclear direction to a concrete one

In the early days, adjustments to the role and function of the MCA councils were required to meet the expectations of the new leadership and to anchor the councils’ role and contribution in the evolving organizational strategy renewal. A common sentiment shared by many co-chairs and members was that the exploratory nature of the early meeting activities made it challenging to know what the intended aims were and what exactly was expected of them, as exemplified by the following quotation:

*The role of the MCAs is one that was quite nebulous initially, and I think everybody was struggling a little bit to get a handle on that. I certainly was, but it has become clearer as we’ve gone through the process what we’re trying to achieve.* (MCA council co-chair)

The H&S team believed the lack of clarity around the MCA councils’ role and purpose was essential to identifying the problem and priorities that would drive the greatest impact. With time, the MCA councils adopted a more concrete direction by being engaged in more action-focused activities with specific, tangible outcomes.

#### Better integrating the voice of PWLE

For some councils, it took some time before PWLE were able to express their messages and share their expertise of the lived experience. With time, PWLE expressed they felt included as equals, more confident and comfortable to speak. This increasing level of comfort and confidence was also perceived by clinician scientists and researchers, and the H&S team. Dialogue became more open and forward, and meetings were increasingly perceived to be a safe space to share ideas and information. With this, a shift occurred where the collective wisdom brought forth by the diverse membership, rather than any one individual’s contribution, was increasingly valued for the greater benefit. The following quotation from one MCA council member exemplifies this:

*We came there very much focused as to our lived experience or research expertise or whatever. But I think that all of us now, if you’re talking about the learning, have a more macro view of what stroke is about and what the priorities should be and we came, we were pretty much focused as to who, for who we’re doing this. It’s for the people. So it’s very much people-centred at the end*. (MCA council member)

By the second year, MCA council members reported seeing higher levels of engagement and integration of PWLE within the larger group. One member noted, “*By year 2 there was no division, it was just coming together as a group, and so we saw a lot of engagement.”* The MCA council structure and process was an awakening to the fact that these hierarchical power structures can be dissolved, resulting in richer conversations and processes, all working towards common goals. The H&S team recognized the processes’ value:

*Comparing the first face-to-face evaluation to the second year face-to-face evaluation, one of the big themes to come up is people feel a lot more comfortable and confident to speak up and they felt it was a safe space to talk. That comes from researchers, from different disciplines or people in sort of different positions.* (H&S team member)

#### Increasing cohesiveness within and across MCA councils

The MCA councils were initially created as six distinct and separate groups representing six individual heart and/or brain conditions to recognize the specialized technical and knowledge areas required to understand and appreciate the uniqueness of each condition. Yet, separating the MCA councils in this way risked further perpetuating silos commonly seen in medicine. To mitigate this risk, from the very beginning of the MCA council journey, the H&S team deemed it important to deliberately acknowledge the silos while keeping a focus on bridging the gaps between them and identifying their commonalities.

H&S leadership made a conscious effort to bridge the gaps between the councils by launching activities (e.g. mock debates, ranking all emerging themes in the presence of all MCA councils) that would permit the identification of the common big questions where H&S could invest its efforts in the coming years to make a difference in the lives of Canadians, regardless of the condition. The following excerpt illustrates the shared sentiment of this deliberate effort amongst council members, which resulted in the intended impact:

*Well, I think it widened even more our views because I think one of the learnings we had, every one of us, were that there were so many similarities in between the different councils, and there were so many transversal things. We were pretty much saying all the same, so in the end, the condition doesn’t really matter that much when you focus on the person.* (MCA council member)

This cross-disease/condition work was ultimately incorporated into CoMPASS, where the crosscutting priorities were eventually recommended and shared with the H&S Board of Directors for approval.

### Challenges resulting from this approach to engagement

There were a number of challenges related to managing, resourcing and terminating the MCA councils. Smaller, logistical challenges arose in the first year of operation, as expressed by members in mid-course evaluations. The H&S team responded promptly to this feedback, making the necessary changes which built trust, transparency and credibility in the H&S leadership team and in the MCA council process. Other challenges were more difficult to resolve completely; either they were too complex to solve within the 2-year mandate, or they only arose after the councils were disbanded.

#### Managing the councils and their membership

The H&S team expressed that identifying members for the councils while ensuring diverse representation was a difficult task. This was especially difficult as time went on, and the councils experienced attrition, including at the co-chair level. Meeting absenteeism was an issue. The overall challenge of “stakeholder relationship management” was felt across all councils. In particular, members described not feeling engaged, despite the fact that the H&S team and co-Chairs invested a great deal of energy in trying to maintain the engagement via one-to-one communication.

A few participants described the balancing act of recognizing and integrating lived experience and legitimizing emotions expressed by PWLE during scientific deliberations. This required careful facilitation. Although people were respectful, that is, expressed care and support, and offered time and space for those who showed emotion, there were hints from some participants that an unintended consequence was that the voice of research was downplayed.

…*the legitimacy of that kind of experience and conversation makes it very, very difficult to actually repudiate the point or offer the contrasting point because the data is personal, and when something is personal, it can be emotional, not inappropriately, it just is, and my fear is and I participate in all of the hours of deliberation, but my fear is that what happened is that some of the risk taking in the robust exchange among experts, sometimes detached from emotion, was actually the volume was toned down significantly.* (Key informant 3)

#### Lack of organizational structure in place to support the initiative

The H&S team spent considerable resources supporting the councils. Meetings were prepared in detail, the needs of PWLE were considered on an ongoing basis, and multiple one-on-one conversations were had to ensure that co-chairs and members were well informed and supported. What was unanticipated, however, was the intensity of the resources required. What emerged from the analysis was that there was an end goal, but a limited, clearly defined process to get there. An H&S member describes this as a lesson learned:

*I think we were developing infrastructure at the same time as kicking this off, so by the infrastructure I mean as simple as a consistent way to communicate with them, we didn’t have a way to share files with them in an easy way, we used email which we know is challenging … Criteria for prioritization* [of information] *and like how do we do that funnelling that we intended to do without having predetermined what the criteria for funnelling and narrowing down the ideas are going to be. So if we do this again I would suggest we do a little bit* [of work] *before we make the call out.* (H&S team member)

#### Terminating the MCA councils

The wrapping up of the councils was disappointing to some. Despite the communication about the closing of the MCA councils, celebration of the successes and the articulation of next steps, some members felt like there was unfinished business. This might have been in part because many outcomes were not immediately tangible.

*So for instance when I look at the outputs, the outputs are very conceptual and they’re all very granular. We all have to do stuff, and so some get stuck too bound down in the mission. When I look at the goals that are up there, they’re too conceptual. Something in there needs to say this is what we’re going to do. It says these are the things that are important, but it doesn’t say this is what we’re going to do and that’s what the third year could have been, and quite frankly I think with more direction out of the gate we could have got to more.* (MCA council co-chair)

Even after its termination, the work continues for H&S. A participant noted that the real timeline to measure the impact of the councils is 5–10 years from now.

### Outcomes arising from this approach to engagement

**Fig. 1:**
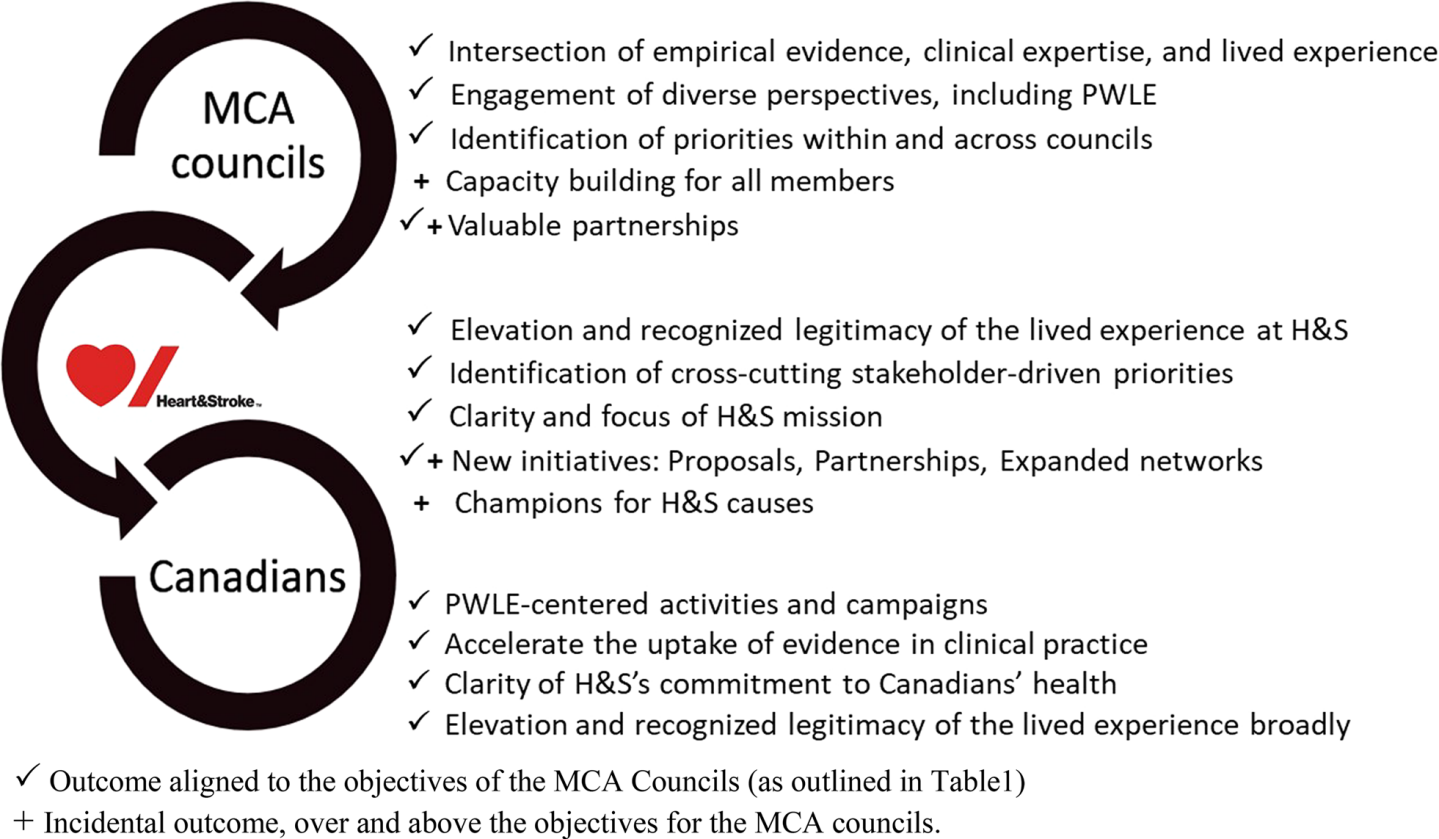
Achieved outcomes of the MCA councils. ✓ Outcome aligned to the objectives of the MCA councils (as outlined in Table1).  + Incidental outcome, over and above the objectives for the MCA councils

Outcomes were achieved at multiple levels: the MCA councils, H&S and at the level of Canadians (Fig. 1).

#### MCA councils

The MCA councils were perceived by all year 2 participants as a resounding success. Within this co-leadership model, empirical evidence, clinical expertise and lived experience intersected. The engagement of diverse perspectives including PWLE was achieved to identify crosscutting priorities within and across councils.

Individual PWLE involved in the process acknowledged this process as a rare and satisfying opportunity to learn more about their condition.

*She said that she didn’t understand her condition until this council met. But she didn’t understand the implications of it until she could talk to researchers and clinicians, and so certainly the influence of people with lived experience in driving strategic directions is like an obvious given for me, but to understand that co-leadership and how it also impacted the understanding of people with lived experience on their own conditions, was a really interesting revelation for me.* (Key informant 4)

Valuable partnerships were built between MCA council members. H&S documents showed that the MCA councils selected the heart–brain connection as top priority. As a respondent noted,

*The involvement of many of the researchers in the MCA councils, first of all it’s created all kinds of collaborative links that didn’t exist before like we’re working on our Stroke Month report and we are developing a partnership with a data scientist, who’s going to help us do our data initializations. If he had not been part of the MCAs, this never would have happened. It’s going to have knock on effects in terms of research publications, but also knock on effects in terms of public awareness and our campaign.* (H&S team member)

#### H&S

H&S’ intended objectives for the MCA councils, to promote engagement and dialogue among PWLE, clinician scientists and researchers, and to provide advice into H&S’s strategic renewal process, were achieved. This resulted in an elevation and recognized legitimacy of the PWLE voice in H&S organizational strategic planning and mission priority-setting.

*Over time, I think they’ve had a great opportunity to give voice to people with lived experience as well as really helping guide the mission work in an informed way and prioritizing our work. It definitely helped inform the strategic renewal process and kind of helped push our boundaries around thinking about kind of where the mission deemed the strengths were and where we would best have impact on our mission work.* (Key informant 4)

MCA council activities and prioritization exercises led to the identification and definition of crosscutting, stakeholder-driven priorities shared by all six MCAs (Table 5). These priorities were endorsed by the Board of Directors to inform and guide the H&S’s mission focus for the next 5 years. These priorities added much-needed clarity and focus to the organization’s mission, which was perceived by respondents as beneficial on multiple fronts, from focusing on the types of research to be funded to marketing and messaging for the purpose of raising public awareness and fundraising.

*I don’t think I realize just how punitive it [the previous lack of clarity] was in terms of both messaging and fundraising, so I think galvanizing these experts to take the thinking beyond the buckets of the disease so to speak and up to a higher level and then getting alignment and focus, I mean I think that’s an amazing thing, and there’s so much opportunity there. I can bet the Heart & Stroke is better off now in terms of mission focus and a galvanized community than it’s ever been, so overall I think this process has been excellent.* (Key informant 2)

H&S used the priorities identified by the MCA councils to inform additional initiatives, over and above the original aims for the MCA councils. From these incidental initiatives, proposals were developed, partnerships created, networks expanded and additional financial resources secured. Some members, inspired by their time on the MCA councils and wanting to further their roles and involvement, are moving on to H&S championing roles for specific causes (e.g. volunteering as part of public awareness campaigns, national events or moving into lead community representative roles on funded cardiovascular research). An example of a formalized and financed partnership that was built is between H&S and the Brain Canada Foundation to launch a team-based, multidisciplinary, multi-institution, multimillion research award emerged from the MCA council process and recommendations.

#### Canadians

Participants predicted that the outcomes from MCA council activities will also have an impact at the population level. The emerging PWLE-centred activities and campaigns (e.g. research, education, policy and advocacy) resulting from the priorities, initiatives, partnerships and resources secured are resulting in outputs intended to accelerate the uptake of evidence in clinical practice and policy to improve outcomes for people at risk of disease and those living with their disease. In addition, according to several MCA council members, the clarity and focus gained through the MCA council process regarding H&S’s mission, the six MCAs and the priorities identified across them is considered an opportunity to clearly communicate H&S’s commitment to Canadians.

*I believe that it also indicates how important it will be to communicate to the general public more about what Heart & Stroke does, the foundation does for research and supporting the health of everyone*. (MCA council member)

The legitimacy of the lived experience and community voice also led to greater attention to the value and importance of patient-oriented outcomes research. To some degree, the integration of PWLE in setting priorities influenced how the relevance of proposed research priority areas in the research funding competitions will be judged.

Taken together, the MCA council process generated greater than expected outcomes at more levels.

*To have gone from a statement where we’re all doing this confusion to where we are today has been quite remarkable, I think and we saw it today with all of the groups, that there’s a sense of we accomplished something. We accomplished something important, and we accomplished something that Heart & Stroke can move forward with.* (MCA council co-chair)

## Discussion and implications

We aimed to understand how advisory councils composed of clinician scientists, researchers, health system leaders, and community members including PWLE, work to inform organizational mission priorities. The intended contribution of this paper is to advance the practice and science of knowledge co-creation and engagement in organizational strategic planning and priority-setting, by understanding the issues, challenges and outcomes arising from this approach to engagement from the perspectives of those included in the process (i.e. PWLE including patients, caregivers and families, researchers, clinician scientists, H&S team and other stakeholders). Our findings lead us to three main points of discussion with implications for three distinct audiences: (1) the index organization (H&S), (2) other organizations interested in this novel approach and (3) researchers interested in knowledge co-creation and engagement models.

Formally involving PWLE as co-leaders and members of the MCA councils left a decisive effect on the culture at H&S. Within this co-leadership model, lived experience, empirical evidence and clinical expertise intersected. This intersection resulted in an unprecedented elevation and recognition of the legitimacy of the PWLE voice in H&S’s organizational strategic planning and mission priority-setting. In the end, this approach impacted organizational values, practices and the incorporation of the PWLE voice in other committees across H&S. This has increased the relevancy of H&S’s work to Canadians, and as a result, enhanced H&S’s ability to move the organization into the future. These findings are particularly relevant in light of calls for a culture change [20–22], or paradigmatic shift [23], to authentically engage patients and members of the public in research, development and organizational activities. The work and reignition of a focus on community members achieved by the MCA councils reveals two opportunities for H&S to leverage. First, a window has opened in terms of improving the public’s awareness of H&S activities. Some work has already begun through their public awareness campaigns and the building of infrastructure to connect those with lived experience with each other. Second, H&S can demonstrate leadership by creating more spaces within the organization for the authentic inclusion of PWLE voices in advising or decision-making.

H&S developed a successful, time-limited, innovative, interactive process and structure that can be adopted by other organizations seeking to include and partner with patients, caregivers and community members in guiding their strategic directions. The key differences between existing, highly structured patient and public engagement approaches, such as the James Lind Alliance (JLA) Priority Setting Partnerships [24], is that the H&S process (1) included diverse representation from leading researchers, clinician scientists, health system leaders, and community members including PWLE, over and above the patients, carers and clinicians’ perspectives required of the JLA approach [24] and (2) engaged PWLE as partners and co-leaders, which is the highest level of engagement on the continuum of engagement [25]. There is limited literature examining partnership with PWLE on advisory boards, councils or committees. More commonly studied is the engagement of patient, family and/or community-only advisory councils (PFACs) [26, 27], which are different from the MCA councils, as they are composed exclusively of PWLE. Hence, what is known from existing studies reporting on the power dynamics, interactions between members, and decision-making structures occurred under different conditions from those of the MCA councils. While our findings reflect many aspects reported in these studies (e.g. power dynamics, participant fatigue, co-creation training for members, and organizational commitment) [28–30], the existing patient engagement literature is heavily focused on the barriers, facilitators and outcomes of this approach [28, 31–33]. Rarely has it focused on the execution or followed the evolution of the partnership over time as our investigation has. Our study further demonstrates how the process can evolve under shifting organizational priorities and how these can be managed. Interested organizations ought to consider a number of investments. First, the success of this initiative was greatly attributed to the organizational and leadership commitment of the H&S team, including the required resources and a steadfast commitment to managing stakeholder relationships. Second, ensuring a diversity of perspectives was a pillar to the process. Having co-chairs representing two different perspectives facilitated openness, shared power and mutual respect. A variety of clinical expertise (medicine, nursing, rehabilitation) and diversity of ethnicity, geography, career stage and context (e.g. system and community leaders) brought varied points of view to the discussions. Although MCA council members felt they were in a safe space, bringing together diverse perspectives led to moments of discomfort and disagreement. Hence, a third investment to consider is an expert facilitator who may help mitigate such discomfort by preparing members to the possibility of personal stories not aligning with the research evidence and ensuring that knowledge from research and community members are equally informative throughout the process. Four, it may be valuable to equip teams and individuals leading and working within PWLE engagement initiatives with skills and competencies to enhance readiness and productive engagement. Training and mentorship opportunities may be useful to enhance lay person communication skills to communicate with the group in a language, tone and manner that is accessible to everyone, including messaging that is personalized, in digestible pieces, and with visual components. For example, the Canadian Institutes for Health Research [22] and the Ontario SPOR [Strategy for Patient-Oriented Research] SUPPORT Unit [34] offer training and resources, many of which are tailored to researchers, patients, clinician scientists or policy-makers. Finally, considerations need to be made for PWLE with physical and cognitive limitations, such as fatigue, and communication disorders such as aphasia, with the understanding that health status and complications could affect ongoing participation [23, 35]. It is imperative that appropriate accommodations and supports are provided to PWLE according to their needs.

Researchers adopting an IKT approach, like we did by working with H&S in this research project, may be interested in our general observations related to the practice of IKT. Our research team grappled with three main tensions throughout the life cycle of this study which are worth noting. First, what we intended to do at the outset of this study differed from what ultimately was achieved. Flexibility and revisions to the original plan were required due to changing leadership at H&S, the evolving nature of the MCA councils, modifications to the MCA councils’ knowledge exchange activities, and COVID-19. We remained in close discussion with the H&S team throughout, staying true to our IKT commitment, and together adapting research activities to the evolving and dynamic context of the H&S and the MCA councils. The adaptations were well supported by the developmental evaluation approach [14], yet impeding them were research ethics board structures and requirements that are not yet fully aligned with IKT approaches. For example, based on the changing context at H&S and how the MCA councils had evolved, we had discussions about modifying data collection tools to better capture the evolution that was taking place. Yet with any significant changes, research ethics boards require amendments delaying study progress until approved, further lengthening an already time-consuming endeavour [36]. Also, in ethics protocols, it can also be difficult to make the distinction between IKT teams that consist of non-researchers contributing to the conduct of the study and who are also study participants from whom data is being collected, and IKT teams that consist of non-researchers contributing to the conduct of the study but who are not study participants. In terms of authorship on products resulting from this work, the research team recognized the knowledge users (in this case, H&S) as coauthors. It was the knowledge users’ preference that the organization, H&S, be listed as a coauthor, recognizing the equally important contributions of all H&S team members involved in the project. Second, the modifications to the study protocol brought forth some discussion on the impact of these deviations to the original research plan on scientific rigour, not unlike Nguyen et al.’s observation that collaborative research orientations are the norm for studies using an IKT approach rather than fixed research methods [37]. We maintain that rigour was not impacted by this, as the H&S team was not involved in the recruitment of participants for data collection, was unaware of who participated, with the entirety of the data analysis left to the research team. Had the research been conducted retrospectively, this would have been avoided, as we would have known what data were available for use, yet the value of being able to capture participants’ experiences in real time cannot be underestimated [38]. We remained sensitive to the potential for participant fatigue, carefully considering the timing and integration of research activities alongside MCA councils’ activities.

## Strengths and limitations

The strengths and opportunities offered by Stake’s case study approach using an IKT lens allowed us to explore, deeply, the MCA council experience from the various perspectives of those involved within the H&S context. We used multiple strategies to enhance the validity of our findings. At the outset, we explicitly disclosed our research team’s positionality and potential research biases. We triangulated data sources and had at least four research team members involved in data collection, analysis and interpretation. We presented year 1 findings to MCA council members and the H&S team as a form of member-checking. The participatory approach to the research meant that the H&S team was involved in every step of the research process. There was a proportionally modest representation of MCA council members and co-chairs which may have led to some perspectives not being captured. Given the prospective nature of the study and the close collaboration with the H&S team, we recognize the potential for social desirability bias in the participants’ responses.

## Conclusion

This research sought to understand the structure, processes and value of advisory councils, co-led and composed of community members including PWLE, clinician scientists, and researchers, tasked with co-creating advice to inform the H&S organizational mission priorities. Engaging PWLE in organizational strategic planning aimed to ensure the issues that matter most to Canadians are prioritized for research funding and are included in H&S advocacy, awareness-raising and fundraising activities. This collaborative structure and process brings together PWLE with multidisciplinary clinician scientists and researchers and enables co-creation of knowledge, but it requires flexibility, a commitment to stakeholder relationship management, and adequate resources. These findings may be helpful to other charities and funding organizations interested in engaging PWLE, the public and other stakeholders into their organizational activities.

## Supplementary Information

**Additional file 1: Table S1.** Data collection activities mapped to MCA council activities.

## Data Availability

The datasets used and/or analysed during the current study are available from the corresponding author on reasonable request.
