# Supplementary material for: Engaging people with lived experience on advisory councils of a national not-for-profit: an integrated knowledge translation case study of Heart & Stroke Mission Critical Area Councils
Source: Health Res Policy Syst. 2022 May 23;20:56. doi: 10.1186/s12961-022-00863-w (PMC9125970; doi:10.1186/s12961-022-00863-w)
Supplement: Supplementary file 1 — Additional file 1: Table S1. Data collection activities mapped to MCA council activities. [file 12961_2022_863_MOESM1_ESM.docx]

**Additional file 1.**

Table S1. Data Collection Activities Mapped to MCA Council Activities

| **MCA Council Activities** | | **Study Data Collection Activities** | | |
| --- | --- | --- | --- | --- |
| **Month/Year** | **Key Activities** | **Month/Year** | **Participants (n)** | **Method** |
| **Year 1** | | | | |
|  | Creation of 6 MCAs | - | - | - |
| July 2017 | Individual member calls and MCA teleconference calls   - Member orientation (i.e., update on strategy renewal, engagement plan) and engagement of Council of Co-Chairs | - | - | - |
| September 2017 | 1^st^ face-to-face MCA Council meetings (x6)   - In-depth review of fact bases, group exploration of issues and needs, and early prioritization by MCA | - | - | - |
| November 2017 – September 2018 | H&S Team:   - Synthesis and theme identification by MCA in collaboration with Co-Chairs, including emerging cross-cutting themes | July 2018 – November 2018 | MCA Council Co-Chairs n=7^a^  Clinician Scientist or Researcher n=3  Community member n=4 | Interviews |
| September/October 2018 | All MCA Council face-to-face meetings   - Further synthesis, refinement and prioritization of cross-cutting themes with all MCA council members | - | - | - |
| October 2018 | H&S Team:   - Synthesis of discussions, deliberations and refinement of prioritized cross-cutting themes in collaboration with Co-Chairs | - | - | - |
| November 2018 | Council of Co-Chairs meeting/All-Council call   - Collective review and validation of prioritized cross-cutting themes/big questions of our time to be brought to CoMPASS for final validation | - | - | - |
| **Year 2** | | | | |
| December 2018 | CoMPASS face-to-face meeting   - Refinement and final validation of prioritized cross-cutting themes/ big questions of our time for H&S to consider   Council of Co-Chairs meeting   - Final review and refinement of the “priorities of our time” to be recommended to the Board | - | - | - |
| January 2019 | H&S Executive team meeting   - Final review and approval of the “priorities of our time” to be recommended to the Board | January – February 2019 | MCA Council members n=25^b^  Clinician Scientist or Researcher n=15  Community member n=10 | Six focus groups |
| February 2019 | H&S Board meeting   - Approval of MCA Councils/CoMPASS recommendations | - | - | - |
| March-June 2019 | MCA Council meetings (x6)   - Engagement/working session on selected priority initiatives | March 2019 | H&S team  n=4 | One focus group |
| January 2020 | CoMPASS face-to-face meeting   - Engagement/working session on selected priority initiatives - Celebration of accomplishments and closing of the MCA Councils’ mandate | January 2020 | MCA Councils Co-Chairs n=11^b^  Clinician/Researcher n=7  Community member n=4 | - One focus group  - Distribution of PEIRS questionnaire^c^ |
| - | - | February 2020 | H&S team  n=4 | One focus group |
| - | - | February- March 2020 | Key informants  n=4 | Interviews |

^a^ Representing 5 of 6 MCA councils

^b^ Representing 6 of 6 MCA councils

^c^Questionnaire aborted due to COVID

Abbreviations: CoMPASS: Council on Mission: Priorities, Advice, Science and Strategy; H&S: Heart & Stroke; MCA: Mission Critical Area; PEIRS: Patient Engagement in Research Scale
